# The impact of session-introducing mindfulness and relaxation interventions in individual psychotherapy for children and adolescents: a randomized controlled trial (MARS-CA)

**DOI:** 10.1186/s13063-022-06212-0

**Published:** 2022-04-11

**Authors:** Julia Kalmar, Ines Baumann, Elena Gruber, Eva Vonderlin, Hinrich Bents, Andreas B. Neubauer, Thomas Heidenreich, Johannes Mander

**Affiliations:** 1 Psychological Institute, Center for Psychological Psychotherapy, University of Heidelberg, Bergheimer Straße 58a, 69115 Heidelberg, Germany; 2 DIPF Leibniz Institute for Research and Information in Education, Rostocker Straße 6, 60323 Frankfurt/Main, Germany; 3 Department of Social Work, Education, and Care, University of Applied Sciences Esslingen, Kanalstraße 33, 73728 Esslingen, Germany

**Keywords:** Mindfulness, Relaxation, Component study, Therapeutic alliance, Randomized controlled trial, Children, Adolescents

## Abstract

**Background:**

The investigation of mindfulness-based interventions (MBIs) in cognitive-behavioral therapy has greatly increased over the past years. However, most MBI research with youth focuses on structured, manualized group programs, conducted in school settings. Knowledge about the implementation and effects of MBIs in individual psychotherapy with children and adolescents is scarce. To fill this research gap, the “Mindfulness and Relaxation Study – Children and Adolescents” (MARS-CA) is designed. It aims to assess the effects of short session-introducing interventions with mindfulness elements on juvenile patients’ symptomatic outcome and therapeutic alliance in individual child and adolescent psychotherapy.

**Methods:**

MARS-CA is conducted at a university outpatient training center for cognitive-behavior therapy. Short session-introducing interventions with mindfulness elements will be compared to short session-introducing relaxation interventions and no session-introducing intervention to explore their effects on symptomatic outcome and therapeutic alliance. The session-introducing interventions will take place at the beginning of 24 subsequent therapy sessions. We hypothesize that patients’ symptomatic outcome and therapeutic alliance improve more strongly in the mindfulness condition than in the other two conditions and that the mindfulness condition moderates the relationship between therapeutic alliance and symptomatic outcome. Patients and their trainee therapists will be randomized to one of the three treatment arms. Participants aged between 11 and 19 years and having a primary diagnosis of either a depressive disorder, an anxiety disorder, or a hyperkinetic disorder will be included. Therapeutic alliance will be assessed after every therapy session (therapy session 1 to therapy session 24), symptomatic outcome will be assessed before the start of treatment (pre), after the 3rd, the 10th, and the 17th therapy sessions, at the end of treatment (24th therapy session, post), and at a 6-month follow-up. Additionally, mindfulness and mindfulness-related measures as well as demographic data, adherence, allegiance, and therapeutic techniques will be assessed. It is our aim to assess a sample of 135 patients. We will conduct multilevel modeling to address the nested data structure.

**Discussion:**

The study can provide information about how add-on MBIs, conducted by trainee therapists, influence therapeutic alliance and symptomatic outcome in individual psychotherapy in children and adolescents.

**Trial registration:**

ClinicalTrials.gov NCT04034576. Registered on July 17, 2019

## Administrative information

Note: the numbers in curly brackets in this protocol refer to SPIRIT checklist item numbers. The order of the items has been modified to group similar items (see http://www.equator-network.org/reporting-guidelines/spirit-2013-statement-defining-standard-protocol-items-for-clinical-trials/).

**Table:** 

Title {1}	The impact of session-introducing mindfulness and relaxation interventions in individual psychotherapies for children and adolescents (MARS-CA): A randomized controlled trial.
Trial registration {2a and 2b}.	ClinicalTrials.gov, Identifier: NCT04034576 (registered 07/17/19).
Protocol version {3}	Version 5, 26th January 2022.
Funding {4}	Self-funded.
Author details {5a}	JK, IB, EG, EV, HB, JM: University of Heidelberg, Psychological Institute, Center for Psychological Psychotherapy, Bergheimer Straße 58a, 69115 Heidelberg. AN: DIPF Leibniz Institute for Research and Information in Education, Rostocker Straße 6, 60323 Frankfurt/Main, Germany. TH: Department of Social Work, Education, and Care, University of Applied Sciences Esslingen, Kanalstraße 33, 73728 Esslingen, Germany
Name and contact information for the trial sponsor {5b}	University of Heidelberg, Psychological Institute, Center for Psychological Psychotherapy. Bergheimer Straße 58a, 69115 Heidelberg, Germany.
Role of sponsor {5c}	The trial sponsor (Center for Psychological Psychotherapy) provides the infrastructure for MARS-CA and enables its functioning.

## Introduction

### Background and rationale {6a}

Mindfulness has its origins in Eastern—mostly Buddhist—traditions and can be conceptualized as a specific form of attention that is non-judgmental, purposeful, and focused on the present moment [1].

This state of being aware of one’s own experiences in the here and now without being judgmental may support the disengagement from perseverating cognitive activities and the enhancement of effective emotion regulation [2]. It is thus reasonable to expect that patients with mental disorders may largely benefit from mindfulness. Therefore, mindfulness is currently being hyped in clinical psychology, with over 600 publications in scientific journals per year [3]. Evidence for the positive effects of mindfulness-based interventions (MBIs) in group settings on outcome measures of various mental disorders have been provided by a number of meta-analyses ( [4–7]; effect sizes from pre–post –follow-up–designs: *g* = 0.31–0.63 [4–6], effect sizes in contrast to other comparison groups (evidence-based treatment to no treatment/waitlist controls): *d* = − 0.004–0.55 [7], *g* = − 0.07–0.53 [5]). However, the most common form of psychotherapy is individual therapy. Empirical evidence on mindfulness in individual therapy is scarce and restricted to adult populations [8, 9]. Therefore, it remains unclear if mindfulness improves individual psychotherapy with children and adolescents.

In the following sections, we will delineate current applications of mindfulness in adult psychotherapy, in child and adolescent psychotherapy, and in individual psychotherapy. Furthermore, we will describe the impact of therapeutic alliance and mindfulness practice on treatment outcome in children and adolescents.

### Application of mindfulness in psychotherapy

The most prominent applications of MBIs are “mindfulness-based stress reduction” (MBSR; [1]) and “mindfulness-based cognitive therapy” (MBCT; [10]). These group programs consist of several elements such as psychoeducation, different mindfulness interventions (e.g., body scan, mindful walking, breathing space), and general group-related factors [11]. Until now, it is still unclear if the mindfulness interventions of these group programs are the crucial element for patients’ symptom improvement. Current studies that compare groups of patients who have participated in the whole MBCT program to groups who have taken part in the MBCT program without the mindfulness elements provide ambiguous results [11–14]. Only a few studies have compared MBIs with active control groups in clinical settings (e.g., [12, 15]), although the comparison to conventional psychotherapeutic methods is crucial to demonstrate the potential impact of MBIs on clinical populations.

### Application of mindfulness programs in child and adolescent psychotherapy

With regard to younger age groups, both MBSR and MBCT have been adapted for children (e.g.‚ A Still Quiet Place: A Mindfulness Program for Teaching Children and Adolescents to Ease Stress and Difficult Emotions’ [16], Learning to Breathe [17], MBCT for Anxious Children (MBCT-C; [18]). Additionally, multiple other programs with mindfulness elements for children and adolescents have been created [19]. Most of these have been developed for application in school settings and all of these are group programs. With regard to children and adolescents with mental disorders, the most prominent mindfulness program is the MBCT-C program [18]. It consists of 12 sessions with 6 to 8 children, each session lasting 90 min. Sessions are composed of a combination of cognitive therapy and mindfulness interventions [18]. Beside the MBCT-C program, which is primarily designed for children with internalizing disorders, further MBCT adaptations are created for children and adolescents with other mental disorders (e.g., the MYMind program for children with attention-deficit/hyperactivity disorder (ADHD) and autism-spectrum disorder (ASD): 9 sessions with 90 min each session, 4 to 6 adolescent participants; [20–22]).

In sum, to the authors’ knowledge, all existing mindfulness programs for children and adolescents are group programs. Although there exist some adaptations for children and adolescents with mental disorders, the main delivery setting of mindfulness programs in youth are schools.

Meta-analyses including both non-clinical and clinical samples revealed small-to-moderate effect sizes (Becker’s del, a measure for the difference of pre-post effect sizes = 0.2–0.5; *g* = 0.305–0.462) of MBIs on treatment effects [4, 23]. A meta-analysis focused on both clinical and non-clinical randomized trials [24], revealing small positive effects of MBIs on mindfulness, executive functioning, attention, depression, anxiety/stress, and negative behavior (*d* ranging 0.16–0.30; [24]). Taking only RCTs with active control treatments into account reduced the number of significant areas with positive effect sizes to mindfulness (*d* = 0.42), depression (*d* = 0.47), and anxiety/stress (*d* = 0.18) [24]. However, the effect sizes in these areas increased from small effect sizes to moderate effect sizes (with the exception of anxiety/stress), indicating that MBIs are more effective than other treatments [24]. It should be noted that the studies with an active control condition included in this meta-analysis have not been conducted in a clinical setting.

### Mindfulness in individual psychotherapy

Many clinicians apply mindfulness interventions in individual therapy [9] and have already done so during their training [25]. Typically, they apply only single mindfulness interventions and not the whole MBSR/MBCT package [9, 26, 27]. Generally speaking, empirical evidence of mindfulness in individual therapy is scarce and results are contradictory, with some studies showing positive effects on outcome measures [28, 29] and a longitudinal study demonstrating no positive effects [30]. A meta-analysis showed that mindfulness interventions as a stand-alone intervention have beneficial effects on anxiety and depression in healthy subjects [31], thus indicating that mindfulness is potentially an effective add-on in individual therapy.

Furthermore, mindfulness practice may have an impact on other variables, such as therapeutic alliance, which will be elaborated in the next section.

### Impact of therapeutic alliance and mindfulness practice on treatment outcome in children and adolescents

Meta-analyses show that therapeutic alliance is positively related to treatment outcome in child and adolescent therapy, with an effect size of *r* = .14 and *r* = .19, respectively [32, 33]. Hence, it is of clinical importance to find methods to strengthen the therapeutic alliance, which in turn could strengthen symptomatic outcome. One possibility to strengthen symptomatic outcome through therapeutic alliance is to practice mindfulness [34], preferably by both therapist and patient [27]. By practicing mindfulness, the transition from stressful daily life to elevated presence in the therapy session is improved, the conversations between therapist and patient are simplified, and the whole therapeutic process is calmed down [27], which may all contribute to improve therapeutic alliance between therapist and patient. Additionally, on the therapists’ side, mindfulness practice enhances self-acceptance and, consequently, improves acceptance and empathy to others (according to the two-stage model of Kristeller and Johnson [35]). Furthermore, therapists’ practice of focusing the attention on the present moment via mindfulness can also increase alliance, as a stronger attention focus on patients is possible.

### Aim of the present study

Summarizing the previous findings, single mindfulness interventions are already frequently implemented in individual psychotherapy with adult patients [9]. However, empirical evidence is scarce [15, 28, 29], and, to the authors’ knowledge, nothing is known about the effects of single mindfulness interventions in child and adolescent psychotherapy. Additionally, there is only limited knowledge about the (non-)superiority of mindfulness interventions to other interventions such as relaxation exercises [15]. To close this research gap, the present study aims (a) to investigate the effects of single mindfulness interventions on symptomatic outcome and therapeutic alliance and (b) to compare the effects of mindfulness interventions to other, already established interventions in individual child and adolescent psychotherapy.

### Objectives {7}

The main purpose of the study is to investigate whether session-introducing mindfulness interventions (SIMI) in comparison to session-introducing relaxation interventions (SIRI) and no specific intervention at the beginning of individual psychotherapy sessions with children and adolescents between 11 and 19 years of age have a positive impact on the therapeutic process. Session-introducing intervention means that in the first 5 to 10 min of a 50-min therapy session, either a short mindfulness intervention, or a short relaxation intervention, or no specific intervention (but the proceeding as usual) will be conducted. This session-introducing intervention could foster self-acceptance and help to focus attention on the present moment of the session, therefore strengthening the therapeutic alliance and outcome effects. Specifically, we want to investigate if SIMI in comparison to SIRI and no intervention at the beginning of psychotherapy sessions lead to a reduction in psychopathological symptomatology and to an improvement of the therapeutic alliance. Furthermore, we hypothesize that the mindfulness condition moderates the relationship between therapeutic alliance and symptomatic outcome. Before the start of therapeutic treatment, patients will be randomized either to the intervention group, practicing SIMI at the beginning of the therapy sessions (IG-M), or to the active control group, practicing SIRI at the beginning of the therapy sessions (CG-R), or to the control group with no specific interventions at the beginning of the therapy sessions (CG). We will investigate the following hypotheses:

H1) We hypothesize that patients of the IG-M will improve more in symptomatic outcome in comparison to the CG-R and CG.

H2) We hypothesize that both patients and therapists of the IG-M will experience stronger increases of therapeutic alliance across the course of therapy in comparison to the CG-R and CG.

H3) We hypothesize that the mindfulness condition moderates the relationship between therapeutic alliance and symptomatic outcome.

### Trial design {8}

**Fig. 1:**
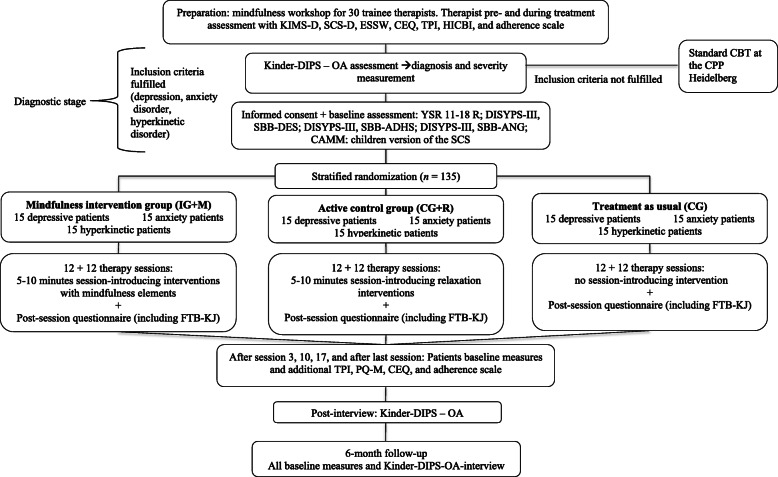
CONSORT flow chart. ADHD, attention-deficit/hyperactivity disorder; CBT, cognitive-behavioral therapy; CEQ, Credibility/Expectancy Questionnaire; CG, control group CG+R, control group: relaxation; CPP, Center for Psychological Psychotherapy; DISYPS-III; SBB-ADHS, Diagnostic System for Mental Disorders; ADHD Self-Rating Scale; DISYPS-III; SBB-ANG, Diagnostic System for Mental Disorders; Anxiety Self-Rating Scale; SBB-DES, Diagnostic System for Mental Disorders; Depression Self-Rating Scale; FTB-KJ, German Version of the Therapeutic Allegiance Scale for Children; HICBI, Heidelberg Inventory of Cognitive-Behavioral Interventions; IG-M, intervention group: mindfulness; Kinder-DIPS – OA, Diagnostic Interview for Mental Disorders in Children and Adolescents – Open Access KIMS, Kentucky Inventory of Mindfulness Skills; SCS, Self-Compassion Scale; TPI, Therapeutic Presence Inventory; YSR, Youth Self-Report

MARS-CA is a single-center, parallel group, three-arm randomized controlled trial. It is designed to test the effectiveness of SIMI in contrast to SIRI to no specific interventions at the beginning of therapy sessions with children and adolescents. Study participants will be randomized either to the intervention group, practicing SIMI at the beginning of the therapy sessions (IG-M), or to the active control group, practicing SIRI at the beginning of the therapy sessions (CG-R), or to the control group with no specific interventions at the beginning of the therapy sessions (CG). A flow chart through the study is shown in Fig. 1.

### Patient involvement in the trial

Juvenile outpatients have assessed all SIMI and SIRI for eligibility prior to the beginning of the trial (65 evaluations from 36 juvenile patients). Subsequently, all SIMI and SIRI have been adapted to the feedback from the patients so that the interventions are appropriate for the implementation in individual psychotherapy with children and adolescents within the trial. No other patient or public involvement has taken place in the presented research design.

## Methods: participants, interventions, and outcomes

### Study setting {9}

The data of MARS-CA will be collected at the outpatient training center for child and adolescent cognitive behavior therapy (CBT) at the Center for Psychological Psychotherapy, Psychological Institute, University of Heidelberg, Germany.

### Eligibility criteria {10}

Inclusion criteria for patients participating in MARS-CA are a primary hyperkinetic disorder, depressive disorder, or anxiety disorder diagnosis. Exclusion criteria for patients are as follows: (a) age below 11 or above 19 years, (b) deficient German language skills, (c) comorbid psychotic disorder, and (d) acute suicidality. Other comorbidities are not considered as an exclusion criterion. Drop-out criteria are (a) termination of individual psychotherapy before the 24th therapy session, (b) refusal to participate in the study any longer, and (c) inability to take part in the interventions any longer (due to severe crises during the therapy process, propensity for dissociation during the interventions, etc.). Trainee therapists who want to take part in the study have to visit two workshops for study preparation.

### Who will take informed consent? {26a}

Voluntary participation and written informed consent by therapists, patients, and their caregivers are necessary conditions for participation in the study. Therapists who want to participate in MARS-CA sign the informed consent before participating in the two study preparation workshops. On patients’ side, the study is typically introduced by a MARS-CA therapist at the first therapy session to the juvenile patient and the caregivers. If the patient shows interest in the participation of the study, both the patient and the caregivers are asked to sign the informed consent. The local ethics committee of the Faculty of Behavioral and Cultural Studies at the University of Heidelberg approved the study protocol in accordance with the Helsinki Declaration.

### Additional consent provisions for collection and use of participant data and biological specimens {26b}

Not applicable, no additional consent provisions for collection and use of participant data and biological specimens in ancillary studies.

### Interventions

#### Explanation for the choice of comparators {6b}

Participants in the control groups will start their therapy sessions either with a relaxation intervention (SIRI, active control condition) or with no session-introducing intervention (non-active control condition). The relaxation exercises have been chosen as comparators as they are a well-established CBT intervention method to reduce inner tension and agitation. No session-introducing intervention has been chosen as comparator because such a non-active comparator will reveal general differences to the active intervention methods.

#### Intervention description {11a}

Depending on the condition (IG-M vs. CG-R vs. CG), three different SIMI, three different SIRI, or no session-introducing intervention will be conducted. With regard to the SIMI, Julia Kalmar (JK) and Johannes Mander (JM) have composed versions for MARS-CA based on the following interventions: mindful walking, body scan, and breathing space of the MBCT-C program by Semple and Lee [18]. During the mindful walking intervention, patients are asked to pace up and down the therapy room and to perceive their movements with an accepting attitude [18]. In the body scan intervention, patients are expected to perceive one part of the body after another in the here and now with reducing the appraisal of thoughts, emotions, and physical sensations to a minimum. The breathing space intervention is some kind of “mini-meditation” during which the patients firstly become aware of their thoughts, emotions, and physical sensations in the present moment, secondly put their focus solely on their breathing, and thirdly extend their awareness beyond their breathing again [18]. The SIRI comprise a self-developed walking relaxation intervention, an adapted version of the progressive muscle relaxation (PMR) for children [36], and an adapted version of the Captain Nemo imagery journey “dolphin riding” [37]. The self-developed walking relaxation intervention has been designed by the authors, together with a child and adolescent psychotherapist having long-term experience in relaxation exercises. It starts with the patients clapping their hands until they have found an appropriate rhythm. In the next step, patients are asked to further clap their hands and to additionally walk through the therapy room, suitable to the clap rhythm. During the PMR intervention, patients are invited to briefly tense and relax single muscle groups in alternation. The intervention ends in tensing and relaxing of all muscle groups of the body [36]. The imagery journey intervention, an adapted version of the Captain Nemo imagery journey “dolphin riding,” consists of an underwater excursion during which the patients imaginatively meet some friendly dolphins [37].

**Fig. 2:**
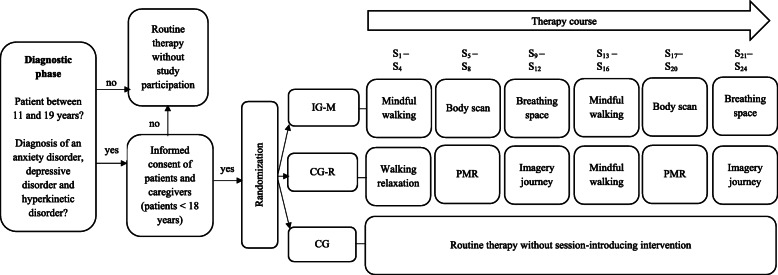
Study procedure of MARS-CA. IG-M, intervention group mindfulness; CG-R, control group relaxation; CG, control group; PMR, progressive muscle relaxation; S, therapy session

All interventions have been assessed for eligibility by a sample of juvenile patients with mental disorders. In the IG-M and the CG-R, each intervention will be instructed by the trainee therapist at the beginning of the first 24 therapy sessions eight times in total, more precisely four times during the first twelve therapy sessions and four times during the second twelve therapy sessions. The interventions of the IG-M and CG-R are parallelized to each other (mindful walking—walking relaxation (length of both interventions: 4 min 15 s); body scan—PMR (length of both interventions: 4 min 40 s); breathing space—imagery journey (length of both interventions: 7 min 30 s)). An overview of the study procedure is depicted in Fig. 2.

#### Criteria for discontinuing or modifying allocated interventions {11b}

Not applicable, no discontinuing or modifying allocated interventions envisaged.

#### Strategies to improve adherence to interventions {11c}

Potential adherence effects are assessed by a self-designed adherence scale.

#### Relevant concomitant care permitted or prohibited during the trial {11d}

Any potential concomitant care will be assessed via questionnaires and will be statistically controlled.

#### Provisions for post-trial care {30}

Not applicable, no provisions for ancillary and post-trial care or compensation to those who suffer harm from trial participation.

#### Outcomes {12}

In the following, the assessment of diagnoses, primary and secondary outcome measures as well as the assessment of demographics, adherence, and therapeutic techniques will be described. For a tabular overview of all measurements, see Table 1.

**Table 1:** Participant timeline of the Mindfulness and Relaxation Study – Children and Adolescents (MARS-CA)

Measures	Before outpatient admission	Diagnostic phase	Pre	S_**1–**_S_**24**_	Mid-S_**3**_	Mid-S_**10**_	Mid-S_**17**_	Post-S_**24**_	6-month follow-up
**Recruitment—therapist**									
Informed consent	+								
**Clinical assessment—therapist**									
HICBI					+	+	+	+	
CEQ therapist					+	+	+	+	
AD-S therapist					+	+	+	+	
**Recruitment—patient**									
Informed consent patient		+							
Informed consent caregiver (patient age < 18 years)		+							
Eligibility screen		+							
Allocation		+							
**Clinical assessment—patient**									
Kinder-DIPS-OA (including severity measurement)			+					+	
YSR 11-18R			+		+	+	+	+	+
DISYPS-III									
SBB-DES			+		+	+	+	+	+
SBB-ANG			+		+	+	+	+	+
SBB-ADHS			+		+	+	+	+	+
CEQ patient					+	+	+	+	
AD-S patient					+	+	+	+	
**Mindfulness and mindfulness-related assessment—therapists**									
KIMS-D			+		+	+	+	+	+
SCS-D			+		+	+	+	+	+
ESSW			+		+	+	+	+	+
TPI therapist					+	+	+	+	
**Mindfulness and mindfulness-related assessment—patients**									
CAMM			+		+	+	+	+	+
SCS (child version)			+		+	+	+	+	+
TPI patient					+	+	+	+	
PQ-M					+	+	+	+	
**Post-session questionnaire—therapists**									
FTB-KJ therapist				+					
TPI (short version) therapist				+					
**Post-session questionnaire—patients**									
FTB-KJ patient				+					
TPI (short version) patient				+					

*Abbreviations*: *AD-S* Adherence Scale, *CAMM* Child and Adolescent Mindfulness Measure, *CEQ* Credibility-/Expectancy Questionnaire, *DISYPS-III* Diagnostic System for Mental Disorders in Childhood and Adolescence, 3rd version, *FTB-KJ* German version of the Therapeutic Alliance Scale for Children, *ESSW* Empathy Scale for Social Workers, *HICBI* Heidelberg Inventory of Cognitive-Behavioral Interventions, *KIMS-D* German version of the Kentucky Inventory of Mindfulness Skills, *PQ-M* Practice Quality – Mindfulness, S therapy session, *SBB-ADHS* problem scales of the attention-deficit/hyperactivity disorder self-rating scale, *SBB-ANG* problem scales of the anxiety disorder self-rating scale, *SBB-DES* problem scales of the depressive disorder self-rating scale, *SCS-D* German version of the Self-Compassion Scale, *TPI* Therapeutic Presence Inventory, *YSR 11-18R* Youth Self Report Revision

#### Diagnoses

Diagnostic Interview for Mental Disorders in Children and Adolescents - Open Access including severity measurements for the main diagnoses [38], according to the ICD-10 [39] and DSM-5 criteria [40], will be applied to establish the diagnoses in patients under the age of 18 years. For patients aged 18 years or older, the Structured Clinical Interview (SCID; [41]) will be applied. All diagnostic interviews will be recorded on video.

#### Primary outcome measures

The primary outcome of the study is patients’ general psychopathological symptomatology (assessed by the total score of the Youth Self-Report 11-18R (YSR 11-18R; [42]). The total score of the YSR-11-18R comprises items related to symptoms of anxiety, depression, somatic complaints, social problems, thought problems, attention problems, rule-breaking behavior, and aggressive behavior [42]. The YSR 11-18R will be assessed before the start of therapy (pre), at the 3rd therapy session (mid-S_3_), at the 10th therapy session (mid-S_10_), at the 17th therapy session (mid-S_17_), at the 24th therapy session (post), and at 6-month follow-up (for more details, see Table 1). Of primary interest are change trajectories of the general psychopathology over the course of therapy. We will assess and compare change trajectories of the three treatment conditions using multilevel-models.

#### Secondary outcome measures

The secondary outcome measures comprise therapeutic alliance, development of mindfulness, self-compassion, and empathy as well as depressive, anxiety, and hyperkinetic symptoms. All questionnaires concerning the secondary outcome measures will be assessed before the start of therapy (pre), at the 3rd therapy session (mid-S_3_), at the 10th therapy session (mid-S_10_), at the 17th therapy session (mid-S_17_), at the 24th therapy session (post), and at 6-month follow-up (for more details, see Table 1).

#### Participant timeline {13}

The participant timeline is depicted in Table 1.

#### Sample size {14}

A recent meta-analysis has detected small to medium effects of MBIs in RCTs with youth in depression (*d* = 0.27), anxiety (*d* = 0.16), executive functions (*d* = 0.30), and attention (*d* = 0.19) [24]. A meta-analysis that has analyzed the effects of meditation in non-clinical groups of adult meditators has detected moderate effect sizes for meditation compared to relaxation (*r* = .21) on psychological outcomes [43].

Taking these results into account, power analyses with G*Power [44] for the detection of small to medium-sized effects (Cohen’s *f* = 0.12) for the interaction between time (pre, T3, T10, T17, T24) and treatment condition (IG-M vs. CG-R vs. CG) (analysis of variance (ANOVA) repeated measures, within-between-interaction, *α* = 0.05, power (1 − β) = 0.80, number of groups = 3, number of measurements = 5, pre–post correlation *r* = 0.5, nonsphericity correction = 1) for the YSR 11-18R total score primary outcome resulted in a sample size of 108 patients. Although this should only be seen as a proxy for the power of the reported multilevel models (MLMs), previous research showed that power in MLM is often comparable to (or even higher than) power in an ANOVA design [45, 46]. The planned sample size should, therefore, be considered a conservative estimate required to detect a small to medium sized condition × time interaction. Taking a drop-out-rate of about 25% into account (i.e., 27 patients), a total sample of 135 patients will be assigned to one of the three treatment groups by a stratified randomization process.

#### Recruitment {15}

The study will be conducted at the outpatient training center for child and adolescent cognitive behavior therapy (CBT) at the Center for Psychological Psychotherapy (CPP) Heidelberg. Approximately 60 trainee therapists treat around 500 patients under 18 years of age, and with different type of mental disorders, per year. More than half of the patients at the outpatient training center are diagnosed with either a depressive disorder, an anxiety disorder, or a hyperkinetic disorder and thus are eligible for MARS-CA. We are optimistic regarding the recruitment process as the trainee therapists and patients are used to research settings due to the university affiliation of the outpatient training center. Additionally, parts of the research team have already successfully completed a similar study with an adult patient sample [15].

### Assignment of interventions: allocation

#### Sequence generation {16a}

Participants in the study will start their therapy sessions either with a SIMI, a SIRI, or no intervention. Treatment allocation will be performed by a blocked and stratified randomization process with a computerized random number generator. Patients will be stratified according to their main diagnosis (hyperkinetic disorder, depression, or anxiety disorder) and then equally randomized into one of the three treatment arms by a balanced blocking procedure.

#### Concealment mechanism {16b}

Randomization lists are saved in a password-protected document on the server of the outpatient training center for children and adolescents of the University of Heidelberg. Only a research assistant of the institute that is not part of the MARS-CA research team has access to this document.

#### Implementation {16c}

Patients will be enrolled in the study by the trainee therapists and in consultation with one researcher of the MARS-CA team. Patients will be assigned to one of the three intervention arms by an independent research assistant who is not part of MARS-CA research team.

### Assignment of interventions: blinding

#### Who will be blinded {17a}

The focus of the current study is on external validity and generalizability of the results to routine clinical practice. Consequently, according to extension of the CONSORT statement concerning the criteria of a pragmatic randomized controlled trial [47, 48], in the current study, no blinding will be implemented.

#### Procedure for unblinding if needed {17b}

Not applicable, no blinding implemented.

### Data collection and management

#### Plans for assessment and collection of outcomes {18a}

MARS-CA trainee therapists who treat MARS-CA patients will conduct the session-introducing interventions and will collect the required outcomes at the appropriate measurement time points (for detailed information about the measurement time points, see Table 1). All MARS-CA therapists will be participating in two workshops on the study process conducted by the research team and prior to the study (workshops content: theoretical information about mindfulness and relaxation interventions, specific study procedure, self-experience of mindfulness exercises between the first and the second workshop). To obtain complete data, trainee therapists are routinely contacted by MARS-CA research assistants and reminded to fill in the required therapist questionnaires and to prompt their patients to do the same. All questionnaires of both therapists and patients are administered via the online platform SoSci Survey (www.soscisurvey.de; [49]) or via paper-pencil.

Primary outcome of the study is the psychopathological symptomatology of the patients. This is assessed by the Youth Self-Report 11-18R (YSR 11-18R; [42]) total score. The YSR 11-18R total score consists of 112 items, which are rated on a three-step scale. The measure shows internal consistencies of .68 ≤ α ≤ .85 for the problem subscales and .88 ≤ α ≤ .94 for second-order scales (external score, internal score, and total score). Convergent and discriminant validity of the YSR 11-18R has been explored with the English version of the YSR in a clinical Australian sample [50]. To the authors’ knowledge, no study has explored the validity of the YSR 11-18R in a German sample. The completion of the YSR 11-18R takes about 15–20 min.

Secondary outcome measures are therapeutic alliance, development of mindfulness, self-compassion, and empathy as well as depressive, anxiety, and hyperkinetic symptoms of the patients. Therapeutic alliance will be assessed by the German version of the Therapeutic Alliance Scale for Children (FTB-KJ; [51]). The FTB-KJ constitutes the session questionnaire, administered to both patients and therapists after every psychotherapy session with a duration of approximately 2 min. The FTB-KJ is a self-report instrument comprising the patient’s as well as the therapist’s perspective, consisting of 12 items rated on a four-step scale and demonstrating good internal consistency (*α* = .83 for the total score). Convergent validity has been demonstrated by significant positive correlations with other instruments to assess therapeutic alliance [51]. Symptom severity has significantly correlated with a poorer therapeutic alliance from the patient’s perspective, especially for boys. Patient and therapist perspective of the FTB-KJ are moderately correlated [51].

General development of mindfulness among patients and therapists across the course of the study will be assessed by the German versions of the “Kentucky Inventory of Mindfulness Skills” (KIMS-D) (therapists; [52]) and the Child and Adolescent Mindfulness Measure (CAMM) (patients; [53]). The KIMS-D consists of 39 items that are rated on a five-step scale and takes about 5 min to complete. Exploratory factor analysis has revealed excellent internal consistencies (.83 ≤ α ≤ .91). Satisfactory convergent and discriminant validity of the KIMS has been demonstrated by significant associations with personality, life satisfaction, and psychopathology questionnaire [52]. The CAMM [53] is based on the KIMS [54] and is intended for youths over the age of 9 years. The measure includes ten items, rated on a five-step scale, with a good internal consistency of *α* = .80. It takes about 3 min to complete. The CAMM has showed significant associations with quality of life, academic competence, and social skills, as well as significant negative associations with somatic complaints, internal, and external psychopathology [53].

Patients’ and therapists’ general development of self-compassion across the course of the study is assessed by the German version of the “Self-Compassion Scale” (SCS-D) (therapists; [55]) and an adapted version of the SCS for Children (patients; [56]). The SCS-D consists of six subscales with 26 items in total and takes about 5 min to complete. Items are rated on a five-step scale. Internal consistency for the total SCS-D scale is *α* = .91. Retest reliability ranges between .72 and .92 [55]. Construct and criterion validity have been shown by positive associations with subjective well-being and negative associations with symptom distress [55]. The SCS for children also consists of 26 items, takes about 5 min to complete, and is rated on a five-step scale. It shows poor to almost excellent internal consistencies with .54 ≤ α ≤ .87 [56]. Construct validity has been given by a positive association between self-criticism and the negative subscales of the SCS for children as well as by a positive association between self-esteem and the positive subscales of the SCS for children [56].

Therapists’ general development of empathy is assessed by the Empathy Scale for Social Workers (ESSW; [57]), adapted for psychotherapists. The ESSW consists of 41 items, rated on a five-step scale, and takes about 5 min to complete. The questionnaire has shown an internal consistency of *α* = .83. Convergent and discriminant validities have been shown by significant positive associations to other empathy scales and a significant negative association with a verbal aggressiveness scale [57].

Patients’ hyperkinetic disorder symptoms are assessed by the problem scales of the ADHD self-rating scale of the Diagnostic System for Mental Disorders in Childhood and Adolescence (DISYPS-III), (SBB-ADHS; [58]), consisting of 20 items rated on a four-step scale, and taking about 5 min to complete. Internal consistency for the total score is *α* = .88. To the authors’ knowledge, no study has explored the validity of the DISYPS-III, SBB-ADHS.

Depressive symptoms of the patients are assessed by the problem scales of the depression self-rating scale of the DISYPS-III (SBB-DES; [58]). The problem scales of the SBB-DES consist of 29 items, rated on a four-step scale, take 5 min to complete, and show an internal consistency of *α* = .89. To the authors’ knowledge, no study has explored the validity of the DISYPS-III, SBB-DES.

Anxiety symptoms of the patients are assessed by the problem scales of the anxiety self-rating scale of the DISYPS-III (SBB-ANG; [58]). The problem scales of the SBB-ANG consist of 44 items, rated on a four-step scale, and take 5 min to complete. Total score consistency is *α* = .94. To the authors’ knowledge, no study has explored the validity of the DISYPS-III, SBB-ANG.

Additionally, demographic data, allegiance effects, and therapeutic techniques will be assessed. As a manipulation check, the Practice Quality-Mindfulness will be applied to patients (PQ-M; [59]). The six items of the PQ-M assess the perceived quality of mindfulness implementation operationalized as perseverance in (a) receptive and (b) present-moment attention. Duration of the questionnaire is about 2 min. The measure shows good psychometric properties with .72 ≤ α ≤ .87. A positive association to another mindfulness questionnaire has confirmed convergent validity. Criterion validity has been given by the predictive power of the PQ-M in relation to measures of life satisfaction and reduction of psychopathological symptoms. The instrument has been developed for adult patients and will be used in an adolescent sample in the present study.

Potential allegiance effects will be assessed by the Credibility/Expectancy Questionnaire (CEQ; [60]). The questionnaire addresses treatment expectancy and rationale credibility in clinical outcome studies. The measure has good psychometric properties with .79 ≤ α ≤ .90 for the subscales and takes about 2 min to complete. Construct validity has been confirmed for the expectancy factor, but not for the credibility factor [60]. It has been developed for adult patients and will be used in an adolescent sample in the present study.

Specific therapeutic techniques will be assessed by the Heidelberg Inventory of Cognitive-Behavioral Interventions (HICBI; [61]). The HICBI consists of 33 items and takes about 5 min to complete. Psychometric properties of the measure are limitedly acceptable to good with .59 ≤ α ≤ .82 and convergent validity is between -.01 ≤ r ≤ .26 for a therapist measure assessing general change. Criterion validity has not been confirmed.

Patients’ and therapists’ in-session therapeutic presence will be assessed by the Therapist Presence Inventory (TPI; [62]). This instrument measures in-session presence operationalized as being fully in the present moment with an attitude of acceptance and openness. Exploratory factor analysis (EFA) has revealed an excellent one-factor structure and good internal consistency with *α* = .75. Duration of the questionnaire is about 3 min. While construct validity is satisfactorily given, criterion validity is only sufficient for the patients’ version, not for the therapists’ version [62]. The instrument has been constructed for adult patients and will be used in an adolescent sample in the present study.

In sum, the completion of the therapists’ survey to each measurement time point takes about 20–25 min, the completion of the patients’ survey to each measurement time point takes about 45–50 min. Additionally, the administration of the session questionnaire after every therapy session takes about 2 min for both the therapist and the patient.

#### Plans to promote participant retention and complete follow-up {18b}

Retention of patients and assessment of data will be realized by close collaboration between trainee therapists and MARS-CA research assistants (via personal meetings, phone calls, and e-mail contact). Assessment of data (during active study participation and follow-up) can be executed either online via an online platform [49] or via paper pencil so that data collection is as convenient as possible for all study participants.

#### Data management {19}

All documents of MARS-CA will be stored at the outpatient training center for children and adolescents of the University of Heidelberg. Data of the study participants will be pseudonymized during active study phase and anonymized after study completion, using a unique study identifier code. With regard to electronic MARS- CA data, all electronic documents will be password-protected. Only MARS-CA staff will have access to them. Informed consent of trainee therapists, caregivers, and patients will be stored separately from other MARS-CA data in a locked cupboard in the MARS-CA research office. All MARS-CA data is stored on the web-based server of the outpatient training center for children and adolescents of the University of Heidelberg. Data of MARS-CA will be stored for at least 10 years after study completion.

#### Confidentiality {27}

All members of the MARS-CA research staff have been informed about data privacy and professional secrecy with regard to MARS-CA participants. Data management will be conducted in accordance to the Data Protection Act, 2018 and in accordance to the demands of the local ethics committee of the Faculty of Behavioral and Cultural Studies at the University of Heidelberg, Germany.

#### Plans for collection, laboratory evaluation and storage of biological specimens for genetic or molecular analysis in this trial/future use {33}

Not applicable, no laboratory evaluation and storage of biological specimens for genetic or molecular analysis in this trial.

### Statistical methods

#### Statistical methods for primary and secondary outcomes {20a}

In line with the recommendations of Baldwin [45], we will apply a multilevel modeling approach to address the nested data structure (sessions at level 1 nested within patients at level 2 nested within therapists at level 3). Thereby, we will treat time as a within-subject (level 1) factor and treatment condition as a between-subject (level 2) factor. Our statistical hypothesis that we expect a stronger reduction of psychopathological symptomatology in the IG-M than in the CG-R and the CG implies a statistically significant interaction effect between group (IG-M vs. CG-R vs. CG) and time. Our hypothesis that the mindfulness condition moderates the relationship between therapeutic alliance and symptomatic outcome implies a significant cross-level interaction between group and therapeutic alliance in predicting (time-varying) symptom severity. Moreover, patients’ and therapists’ pre-treatment characteristics will be investigated as predictors at levels 2 and 3 to control for differential effects on the outcome in the three treatment arms.

#### Interim analyses {21b}

To the present date, no interim analyses are planned. The results of the study preparation workshops for the trainee therapists will be analyzed before MARS-CA termination. Decision to terminate the trial is up to the authors.

#### Methods for additional analyses (e.g., subgroup analyses) {20b}

To the present date, no additional analyses such as subgroup analyses are planned. In principle, additional analyses are possible.

#### Methods in analysis to handle protocol non-adherence and any statistical methods to handle missing data {20c}

According to the revised CONSORT statement, statistical analyses will be conducted by an intention-to-treat as well as a completer analysis [47]. Missing data will be handled according to the most recent guidelines for multilevel analyses.

#### Plans to give access to the full protocol, participant level-data, and statistical code {31c}

The MARS-CA protocol is available for the public under clinicaltrials.gov. Final trial data set and statistical code will be available from the corresponding author upon reasonable request after completion of MARS-CA.

### Oversight and monitoring

#### Composition of the coordinating center and trial steering committee {5d}

The research team of MARS-CA will coordinate and manage the entire study process. It will meet regularly to discuss ongoing study questions of any type.

#### Composition of the data monitoring committee, its role and reporting structure {21a}

Due to the overall low risk of MARS-CA for its participants and the fact that MARS-CA is a rather small one site study, no data monitoring committee is needed.

#### Adverse event reporting and harms {22}

The MARS-CA research team will be in close contact with the trainee therapists who participate in the study to monitor for adverse events of the patients during the session-introducing interventions. If patients report discomfort during the interventions, they are asked to immediately get in touch with their therapists. In turn, trainee therapists are requested to report their patients’ difficulties to the MARS-CA research team. The MARS-CA research team discusses the difficulties delineated and decides if it is justifiable that the patient further participates in the study or not. Any adverse events that lead to a discontinuation of MARS-CA will be documented.

#### Frequency and plans for auditing trial conduct {23}

Not applicable, no independent monitoring and auditing considered.

#### Plans for communicating important protocol amendments to relevant parties (e.g., trial participants, ethical committees) {25}

Every important protocol amendment will be communicated to the ethics committee of the Faculty of Behavioral and Cultural Studies at the University of Heidelberg, Germany. The amendment can only be implemented after its approval.

#### Dissemination plans {31a}

The results of MARS-CA will be published in peer-reviewed articles after study completion.

## Discussion

To date, MBIs with children and adolescents are mostly applied in structured, manualized group settings [4, 23, 24]. Additionally, child and adolescent samples are mainly recruited in school settings, not in clinical settings [19, 63]. However, MBIs are often implemented as add-on interventions in routine individual adult psychotherapy [9], and it can be assumed that this is also the case in individual child and adolescent psychotherapy. It has been shown that stand-alone MBIs have beneficial effects on anxiety and depression in adults [31]. A first recommendation for the application of stand-alone MBIs in individual psychotherapy has recently been published [64]. However, the authors pointed out that more RCTs are necessary to prove the effectiveness of stand-alone MBIs in comparison to other interventions [64]. For that reason, we designed the MARS-CA study to analyze the effects of three SIMI at the beginning of individual psychotherapy with children and adolescents. We will investigate the effects using a RCT under effectiveness conditions similar to a former RCT in individual adult psychotherapy of our workgroup [15]. Three SIRI are used as active control condition, and no intervention at the beginning of the individual child and adolescent psychotherapy serves as passive control condition.

### Strengths of the MARS-CA study

Our study is the first one to investigate the effects of different session-introducing interventions (IG-M, CG-R) on psychopathological symptomatology and therapeutic alliance in comparison to no session-introducing intervention in individual psychotherapy with children and adolescents. MBIs in this age group are almost solely used in structured, manualized group therapy programs and will be applied in individual therapy in this study. As MARS-CA will be conducted at a university outpatient training center for children and adolescents, a bridge can be built between theoretical workshop input, its practical application in individual child and adolescent psychotherapy, and psychotherapy research.

### Limitations of the MARS-CA study

Due to the naturalistic design of MARS-CA, both patients and therapists know about the three treatment arms (IG-M, CG-R, CG) and are not blinded. This issue is addressed according to the revised CONSORT statement [47, 48]. A research assistant that is not involved in the study randomizes the patients to one of the three treatment arms. All researchers and participants of MARS-CA are blind to the randomization process. Additionally, we try to minimize the patients’ and therapists’ bias by assessing the intensity of patients’ active involvement in the interventions during the therapy sessions and at home and by applying an allegiance scale to patients and therapists that addresses positive and negative attitudes towards all interventions. Furthermore, we will apply a crossed-therapist design so that every therapist may treat patients from each treatment condition (IG-M, CG-R, CG). By doing so, therapists’ bias to either mindfulness or relaxation approach will be minimized as therapists most likely treat patients from all study conditions.

## Conclusion

In the present study, MARS-CA, we will compare the impact of SIMI, SIRI, and no intervention on both psychopathological symptomatology and therapeutic alliance in individual therapy with children and adolescents. The results of the study may provide initial evidence on how add-on MBIs may be implemented in individual therapy with children and adolescents without conducting a whole manualized program and on how MBIs affect treatment outcome in comparison to an active and passive control intervention.

## Trial status

The present study protocol is based on protocol version No. 5, 26 January 2022. Recruitment began in April 2019 and will approximately be completed in July 2023.

## Abbreviations

ADHD: Attention-deficit/hyperactivity disorder
CAMM: Child and Adolescent Mindfulness Measure
CBT: Cognitive behavioral therapy
CEQ: Credibility/Expectancy Questionnaire
CG: Non-active control group
CG-R: Active control group with session-introducing relaxation exercises
CONSORT: Consolidated standards of reporting trials
CPP: Center for Psychological Psychotherapy
DISYPS-III: Diagnostic System for Mental Disorders in Childhood and Adolescence
DSM-5: Diagnostic and Statistical Manual of Mental Disorders
EFA: Exploratory factor analysis
ESSW: Empathy Scale for Social Workers
FTB-KJ: German version of the Therapeutic Alliance Scale for Children
HICBI: Heidelberg Inventory of Cognitive Behavioral Interventions
ICD-10: International Classification of Diseases, 10th edition
IG-M: Intervention group with session-introducing mindfulness exercises
KIMS: Kentucky Inventory of Mindfulness Skills
MARS-CA: Mindfulness and Relaxation Study – Children and Adolescents
MBCT: Mindfulness-based cognitive therapy
MBI: Mindfulness-based interventions
MBSR: Mindfulness-based stress reduction
PQ-M: Practice Quality – Mindfulness
RCT: Randomized controlled trial
SBB-ADHS: Attention-deficit/hyperactivity disorder self-rating scale
SBB-ANG: Anxiety self-rating scale
SBB-DES: Depression self-rating scale
SCID: Structured Clinical Interview for DSM-5
SCS: Self-Compassion Scale
SIMI: Session-introducing mindfulness interventions
SIRI: Session-introducing relaxation interventions
TPI: Therapeutic Presence Inventory
YSR: Youth Self-Report
